# A platform for phenotypic discovery of therapeutic antibodies and targets applied on Chronic Lymphocytic Leukemia

**DOI:** 10.1038/s41698-018-0061-2

**Published:** 2018-09-03

**Authors:** A. Ljungars, L. Mårtensson, J. Mattsson, M. Kovacek, A. Sundberg, U-C. Tornberg, B. Jansson, N. Persson, V. Kuci Emruli, S. Ek, M. Jerkeman, M. Hansson, G. Juliusson, M. Ohlin, B. Frendéus, I. Teige, M. Mattsson

**Affiliations:** 1 BioInvent International AB, Lund, Sweden; 2 Department of Immunotechnology, Lund University, Lund, Sweden; 3 Hematology clinic, Skåne University Hospital, Lund University, Lund, Sweden; 4 Present Address: HansaMedical AB, Lund, Sweden; 5 Present Address: Division of Oncology and Pathology, Department of Clinical Sciences, Lund University, Lund, Sweden

## Abstract

Development of antibody drugs against novel targets and pathways offers great opportunities to improve current cancer treatment. We here describe a phenotypic discovery platform enabling efficient identification of therapeutic antibody-target combinations. The platform utilizes primary patient cells throughout the discovery process and includes methods for differential phage display cell panning, high-throughput cell-based specificity screening, phenotypic in vitro screening, target deconvolution, and confirmatory in vivo screening. In this study the platform was applied on cancer cells from patients with Chronic Lymphocytic Leukemia resulting in discovery of antibodies with improved cytotoxicity in vitro compared to the standard of care, the CD20-specific monoclonal antibody rituximab. Isolated antibodies were found to target six different receptors on Chronic Lymphocytic Leukemia cells; CD21, CD23, CD32, CD72, CD200, and HLA-DR of which CD32, CD200, and HLA-DR appeared as the most potent targets for antibody-based cytotoxicity treatment. Enhanced antibody efficacy was confirmed in vivo using a patient-derived xenograft model.

## Introduction

Drug discovery is either phenotypic or target based. In phenotypic discovery, molecules with a desired effect on the phenotype of a cell or an organism are isolated followed by identification of their targets. For small molecules a large fraction of first-in-class drugs have been identified in this way.^1,2^ However, for antibodies, which are the fastest growing class of drugs, only a few have been isolated using phenotypic discovery (for a comprehensive review see Minter et al. 2017^3^).

From a theoretical perspective, antibody-based phenotypic discovery makes particular sense.^4^ It enables, depending on the selected phenotypic assay, both identification of antibodies that mediate their mechanism of action through the biology of the target receptor and through effector mechanisms such as Fc-receptor binding and engagement of immune effector cells. Furthermore, by the use of primary patient cells, phenotypic discovery enables development of personalized drugs.

Phenotypic discovery using contemporary antibody libraries with tens of billions of antibodies does however, pose significant technical challenges. In essence, (1) isolation of 100s–1000s of antibodies that, ideally, comprise specificity for all therapeutically relevant cell surface receptors, (2) high-throughput phenotypic screening of antibodies in clinically predictive in vitro and in vivo assays, and (3) target deconvolution of antibodies.

## Results

**Fig. 1:**
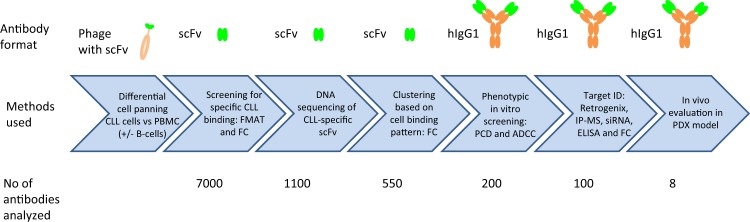
Schematic outline of the methods included and the number of antibodies analyzed in the various steps of the CLL study. A CLL-specific antibody pool was generated by differential cell panning and individual soluble antibodies in scFv format were screened for cell binding in flow cytometry, FC, and fluorometric microvolume assay technology, FMAT. Clones binding specifically to CLL cells were DNA sequenced and unique clones were clustered based on cell binding pattern analysis. Several clones from each cluster were functionally tested in hIgG1 format in PCD and ADCC assays. Targets were identified for a subset of clones followed by in vivo testing in a PDX model

**Fig. 2:**
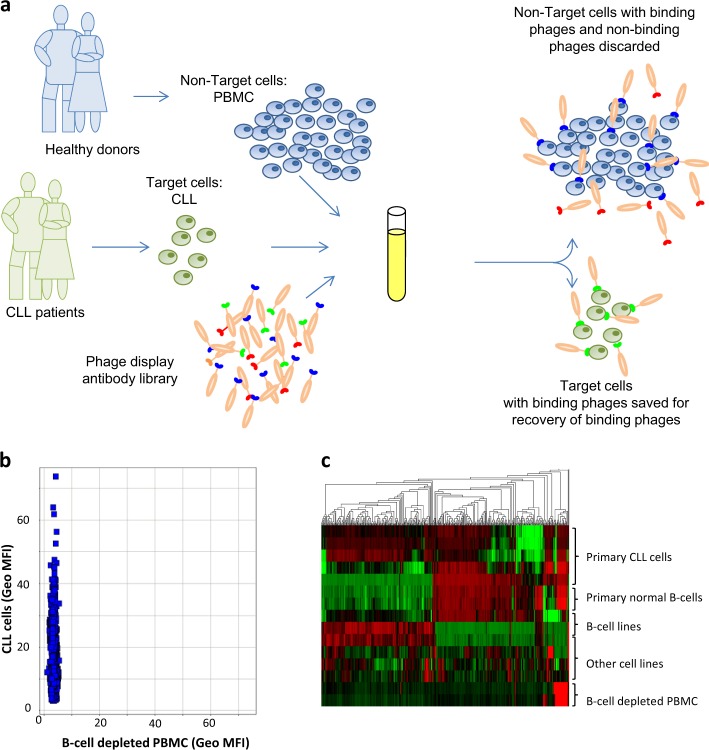
Generation and binding characterization of CLL-specific antibodies. **a** Schematic overview of the phage-display panning procedure. To enrich for CLL-specific antibodies, phages displaying scFv were mixed and incubated with a pool of CLL cells from multiple patients and a pool of PBMC from multiple healthy donors (with or without prior depletion of B-cells). Target (CLL) and non-target (PBMC) cells were then separated and phages binding CLL cells were eluted. **b** Screening in flow cytometry of 1152 scFvs’ binding to CLL cells from one patient versus binding to B-cell depleted PBMC from one healthy donor. **c** Heatmap showing the flow cytometry binding pattern of 392 unique scFvs to CLL cells from 5 patients, B-cells, and CD19-negative cells (B-cell depleted PBMC) from 2 healthy donors, B-cell lines (Raji and RPMI8226) and other cell lines (DU145, Lovo, MCF-7, and HS-5). Based on binding profile a hierarchal clustering of antibodies was made using Qlucore^TM^ Omics Explorer. Clones were color-coded based on relative signal intensities within each cell type where red represents the strongest binding to a particular cell type, green the weakest binding and black in-between

To facilitate phenotypic discovery of antibody drugs we have developed the function-FIRST antibody discovery platform F.I.R.S.T^TM^ here demonstrated in a case study on Chronic Lymphocytic Leukemia (CLL) (summarized in Fig. 1). A pool of CLL-specific antibodies was generated by differential cell panning, applying positive selection pressure to primary CLL cells and concomitant negative selection pressure to peripheral blood mononuclear cells (PBMC) from healthy donors, using the phage-display human antibody library n-CoDeR^®^^5^ (Fig. 2a). PBMC from healthy donors were chosen as non-target cells as they are easy to obtain in large cell-numbers, contain critical immune effector cells, e.g. CD8+ T cells that one may not want to target, and additionally express many general hematopoietic antigens with limited therapeutic potential. Isolated antibody fragments (scFv format) showed high selectivity for the target cells; ~1100 out of 7000 clones bound CLL cells but not PBMC from healthy donors (Fig. 2b). DNA sequencing of genes encoding CLL-specific scFvs resulted in 550 unique sequences (at least three amino acids difference in the CDR regions) of which 500 had a unique CDRH3, demonstrating a high variability in the generated antibody pool. Unique scFvs were analyzed by flow cytometry for binding to primary patient CLL cells, various cell lines, and PBMC from healthy donors. Clustering of scFvs based on cell binding specificity demonstrated several distinct binding patterns, strongly suggesting antibody binding to a panel of different targets (Fig. 2c).

To enable functional studies, several antibodies from each specificity cluster were produced as full length human IgG1 (in total ~200). Two functional assays were selected for phenotypic screening, programmed cell death, PCD, representing direct killing of target cells (Supplementary Fig 1a, b) and antibody dependent cellular cytotoxicity, ADCC, representing immune effector cell mediated target cell death (Supplementary Fig 1c, d). Each antibody was tested on CLL cells from two or more patients and around 20 and 15% showed improved PCD (Supplementary Fig 1e) or ADCC (Supplementary Fig 1f), respectively, compared to the benchmark, the anti-CD20 antibody rituximab, which is often used in combination with chemotherapy as first-line treatment of CLL.^6,7^

A subset of IgGs (including both positive and negative clones from the phenotypic screening) was analyzed to determine target specificity using a combination of three methods; binding to transfected cells in a chip based format (Retrogenix), immunoprecipitation followed by mass spectrometry analysis, IP-MS, and reduced binding to cells after siRNA transfection. The specificity of most antibodies was determined and altogether six targets, CD21, CD23, CD32, CD72, CD200, and HLA-DR, were identified (Supplementary Table 1). Antibody specificity was confirmed by two techniques, ELISA (Supplementary Fig 2a) and flow cytometry (Supplementary Fig 2b). ELISA showed that high affinity antibodies were isolated with EC50 values in the low nM range.

All 550 unique antibodies were subsequently analyzed for binding to the identified targets in ELISA. Adding target identities into the antibody binding heat map showed that antibodies with the same specificity have a distinct cell binding profile and group together (Supplementary Fig 3 and 4).

**Fig. 3:**
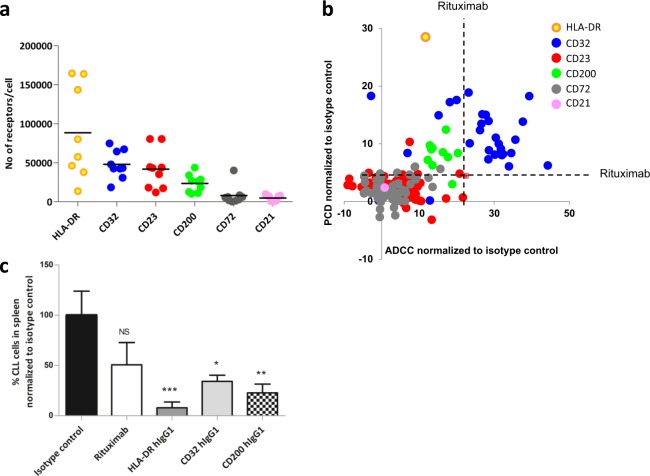
Target protein expression levels on CLL cells and in vitro and in vivo functional data. **a** Number of receptors for identified targets HLA-DR, CD32, CD23, CD200, CD72, and CD21 on CLL cells (*n* = 9 patients except HLA-DR *n* = 8). Cells were stained with commercially available fluorescence labelled antibodies against the identified targets at binding saturated concentrations and analyzed by flow cytometry. The number of receptors per CLL cell was calculated based on parallel staining and analysis of calibration beads. **b** Correlation between target identity and in vitro functional data. Results from PCD and ADCC assays were plotted against each other and color-coded based on antibody specificity. **c** Screening of antibodies (hIgG1) in a PDX model using CLL cells from a rituximab-resistant patient, showing the fraction of CLL cells in spleen (normalized to isotype control) after antibody treatment (mean with SEM for *n* = 8 mice/antibody except isotype control were *n* = 7). For statistical analysis all antibodies were compared with the isotype control antibody (one-way-ANOVA) using GraphPad Prism

Differentially expressed surface receptors suitable for antibody therapy may be expressed at different absolute levels on target and non-target cells, here CLL cells and PBMC from healthy donors, making it important that an antibody selection process can identify binders to antigens expressed at substantially different levels. To define the platform’s potential in this respect, the number of antibody-target receptors per cell was determined. CD23, CD32, CD200, and HLA-DR are highly expressed on CLL cells (30,000–90,000 receptors/cell) whereas CD21 and CD72 are present in low numbers (4000–8000 receptors/cell) (Fig. 3a, Supplementary Table 2). All target receptors are expressed in very low numbers on PBMC without B-cells (non-detectable to 2000 receptors/cell).

Mapping of anti-CLL cytotoxicity (PCD and ADCC) as a function of target specificity revealed that CD32, CD200, and HLA-DR have the greatest functional potential with several antibodies showing improved efficacy compared to the benchmark rituximab (Fig. 3b). With a few exceptions, antibodies targeting CD21, CD72, and notably CD23, despite being higher expressed than CD200 and at a similar high level as CD32, showed no added positive effect in the selected phenotypic assays. Variation in functionality was additionally seen within each group of antibodies binding a specific target (Fig. 3b). One explanation could be that these antibodies target different epitopes. Importantly, this demonstrates the strength of the phenotypic screening which enables identification of optimal antibody-target combinations.

A subset of generated antibodies (in total 8) specific for targets associated with optimal PCD and ADCC (CD32, CD200, and HLA-DR) was further evaluated in a patient-derived xenograft, PDX, model (Supplementary Fig 5a). Fresh human CLL cells, injected into mice, home to and proliferate in lymphoid organs.^8^ CLL cells in the spleen retain the tumor’s sensitivity or resistance to rituximab (Supplementary Fig 5b) indicating a potential of this model to assess treatment responses of tumors sensitive and resistant to rituximab-based therapy. All tested antibodies depleted CLL cells from rituximab-responder patients and CLL-depleting antibodies to all three targets were identified also using CLL cells from rituximab-resistant patients (Fig. 3c). The phenotypic discovery process thus identified several antibody-target combinations with improved in vivo effects compared to the anti-CD20 antibody rituximab.

## Discussion

We have developed a phenotypic discovery platform enabling use of primary cells throughout the discovery process (panning, screening, and functional studies) in contrast to phenotypic antibody discovery efforts described by others where either cell lines^9^ or cultivated primary cells^10,11^ have been used. Applied on CLL cells we successfully isolated first-in-class antibodies with improved efficacy compared to rituximab. In particular, the phenotypically discovered cytotoxic activity of anti-CD32 antibodies on CLL cells disclosed CD32 as a potential target for CLL treatment. Based on this finding and background knowledge on CD32 receptors, we have in a separate effort isolated a CD32b-specific antibody, BI-1206.^8^ This antibody is currently tested in two phase I/II clinical trials (NCT02933320 and NCT03571568) on CD32b-positive B-cell lymphomas and leukemias. Thus, phenotypically discovered targets can be translated to the clinic.

A useful phenotypic discovery platform should be able to select binders against a diversity of targets, not only the most abundant membrane proteins. Importantly, the differential panning procedure described here enabled discovery of antibodies against differentially expressed receptors present at both high and low levels on the surface of the target cells. The increased number of targets identified here compared to others^9–11^ indicates that our platform generates a higher target diversity.

Phenotypic, in contrast to target-based, drug discovery requires downstream identification of targets engaged by the identified antibodies. Such target deconvolution is typically a difficult and labor-intensive step. We demonstrated that cell binding heat map analysis can be used to group antibodies with the same specificity together. This approach facilitates the target deconvolution process, as it allows us to limit the number of analyzed antibodies that have to be investigated in low throughput, high-cost techniques, such as IP-MS. Instead specificity, in many cases, can be confirmed through testing in a high throughput, cost-effective technique, such as ELISA.

We are confident that the platform can be expanded to other cell types including individual patient cells, enabling development of personalized antibody drugs. We also foresee that it can be used for identification of antibodies that synergize with existing therapies and thereby has the potential to improve treatment of many cancer types.

## Methods

An overview of the methods used is shown in Fig. 1. Ethical approvals for human cells were obtained by the Ethics Committee of Skåne University Hospital and written informed consent was provided in accordance with the declaration of Helsinki. Mice experiments were performed in agreement with ethical permissions from Malmö Lund Animal Ethics Committee. Detailed methods are provided as Supplementary Materials and Methods.

### Data availability

The data sets that support the findings of this study are not publicly available due to their commercial value but are available from the corresponding author upon reasonable request.

## Electronic supplementary material

Supplementary Figures and Methods

## Electronic supplementary material

**Supplementary information** accompanies the paper on the *npj Precision Oncology* website (10.1038/s41698-018-0061-2).
