# Supplementary material for: A platform for phenotypic discovery of therapeutic antibodies and targets applied on Chronic Lymphocytic Leukemia
Source: NPJ Precis Oncol. 2018 Sep 3;2:18. doi: 10.1038/s41698-018-0061-2 (PMC6120912; doi:10.1038/s41698-018-0061-2)
Supplement: Supplementary file 1 — Supplementary Figures and Methods [file 41698_2018_61_MOESM1_ESM.pdf]

Supplementary Figure 1

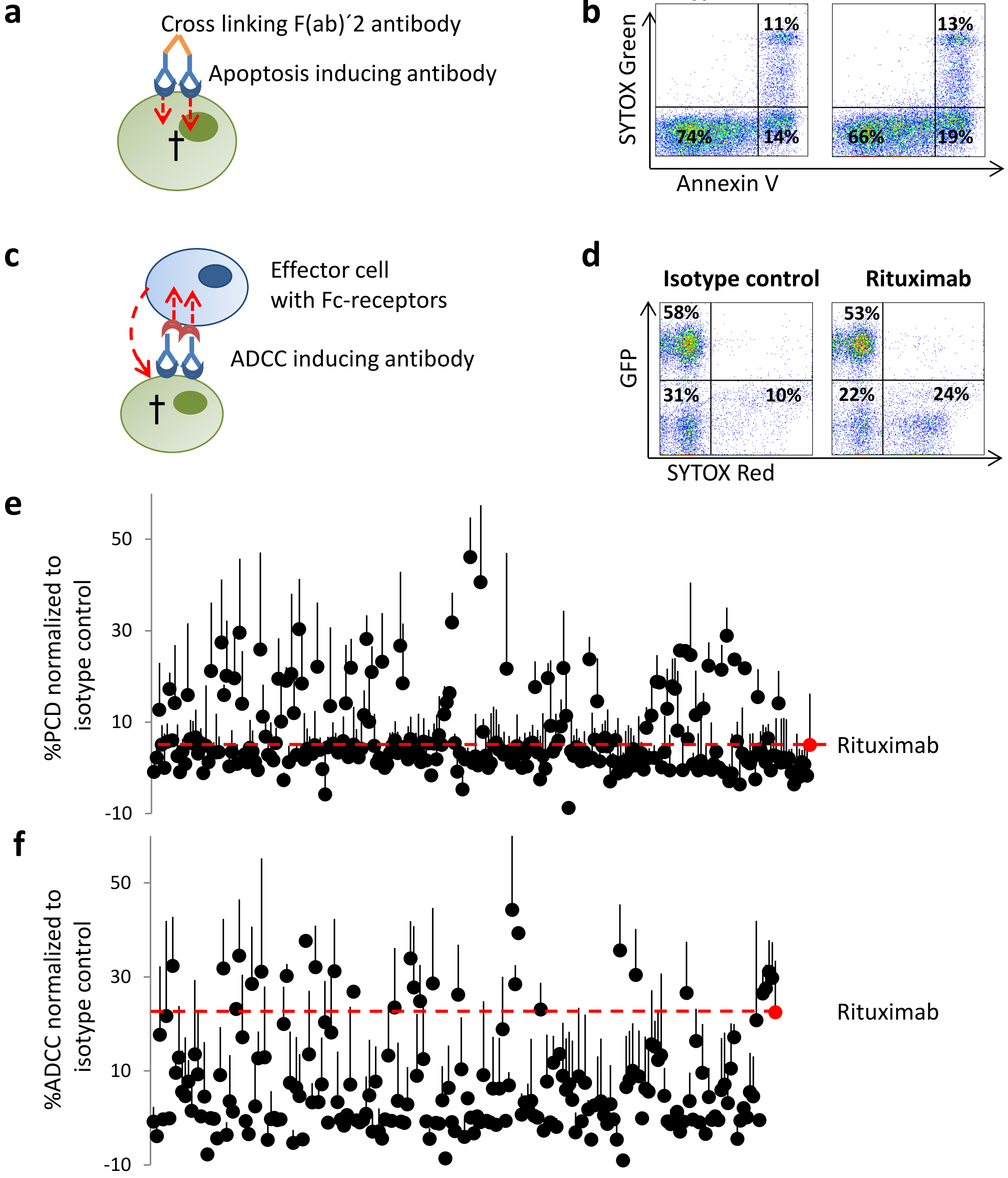

Supplementary Figure 1. Phenotypic screening of antibodies using PCD and ADCC
(a) Schematic outline of the PCD assay, antibodies (hgG1) were added to CLL cells and cross-linked using a polyclonal F(ab)'2 anti-human-Fc specific antibody at 5 times molar excess. After overnight incubation at +37°C, 5% CO_2, cells were stained with Annexin V and SYTOX Green. (b) The fraction of dead cells in the PCD assay was analyzed by flow cytometry as Annexin V^+ cells and here visualized as SYTOX Green vs Annexin V. (c) Schematic outline of the ADCC assay, where a GFP-expressing NK cell line was used. CLL cells, antibodies (hIgG1) and NK cells were incubated for 4 h at +37°C, 5% CO_2 before staining with SYTOX Red. (d) The fraction of dead cells in the ADCC assay was analyzed by flow cytometry as GFP^-, SYTOX Red^+ compared to all GFP^- cells. (e, f) Phenotypic screening of antibodies to analyze their ability to induce PCD (n=251) (e) and ADCC (n=196) (f) with CLL cells from at least two patients using rituximab as benchmark. Data were normalized to an isotype control.

Supplementary Figure 2

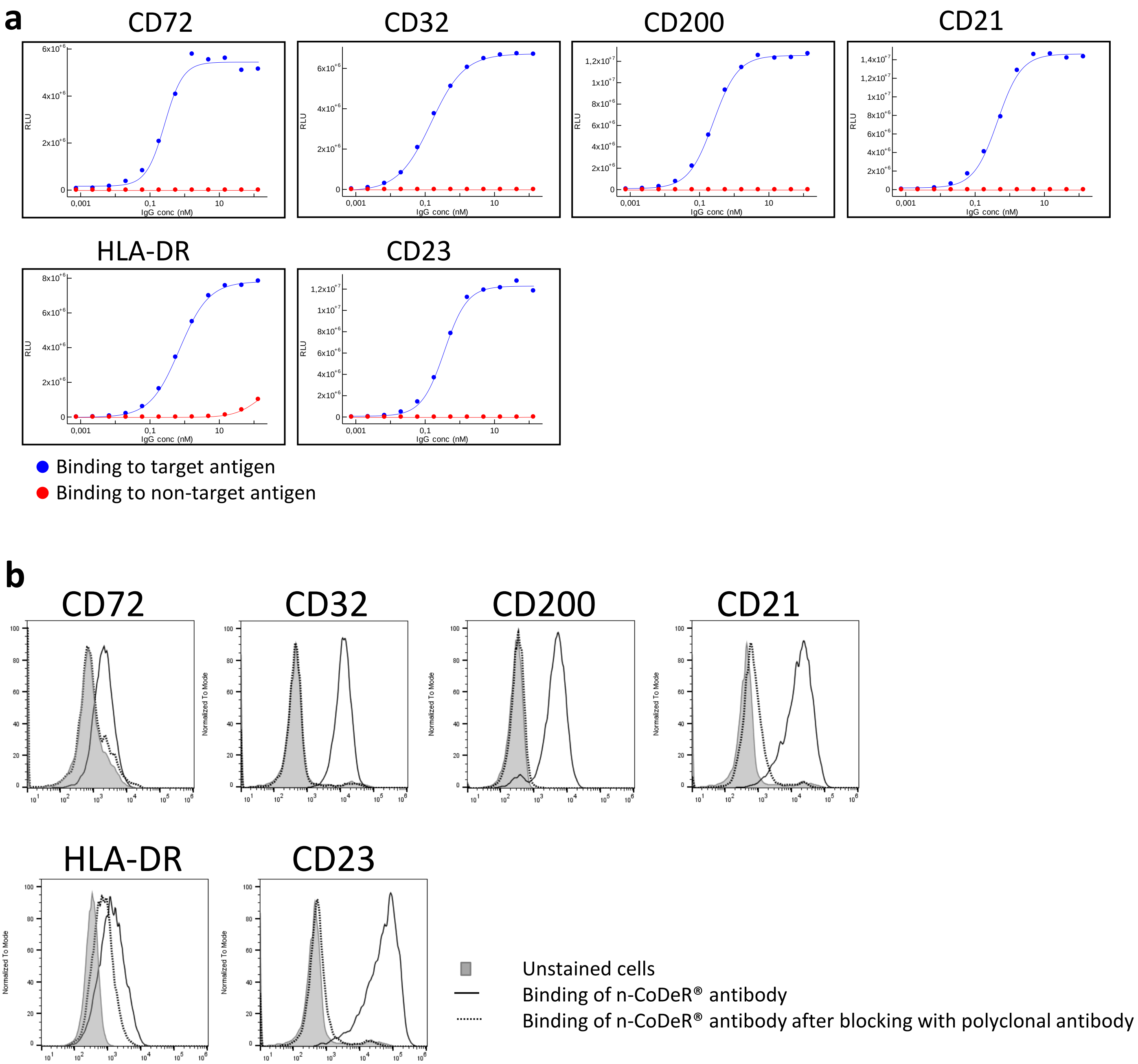

Supplementary Figure 2. Confirmation of antibody specificities

(a) Dose-response ELISA of one representative antibody (hIgG1) per identified target. Each antibody was analyzed against the specific purified target protein and a non-target protein carrying the same tag as the target. Binding was detected using a HRP-labelled anti-human antibody and a luminescent substrate. (b) Flow cytometry analysis showing the binding of one antibody (hIgG1) per target to Raji (all targets except CD200) or CLL cells (CD200) with or without previous blocking with a target-specific monoclonal (HLA-DR) or polyclonal (all other targets) antibody.

Supplementary Figure 3

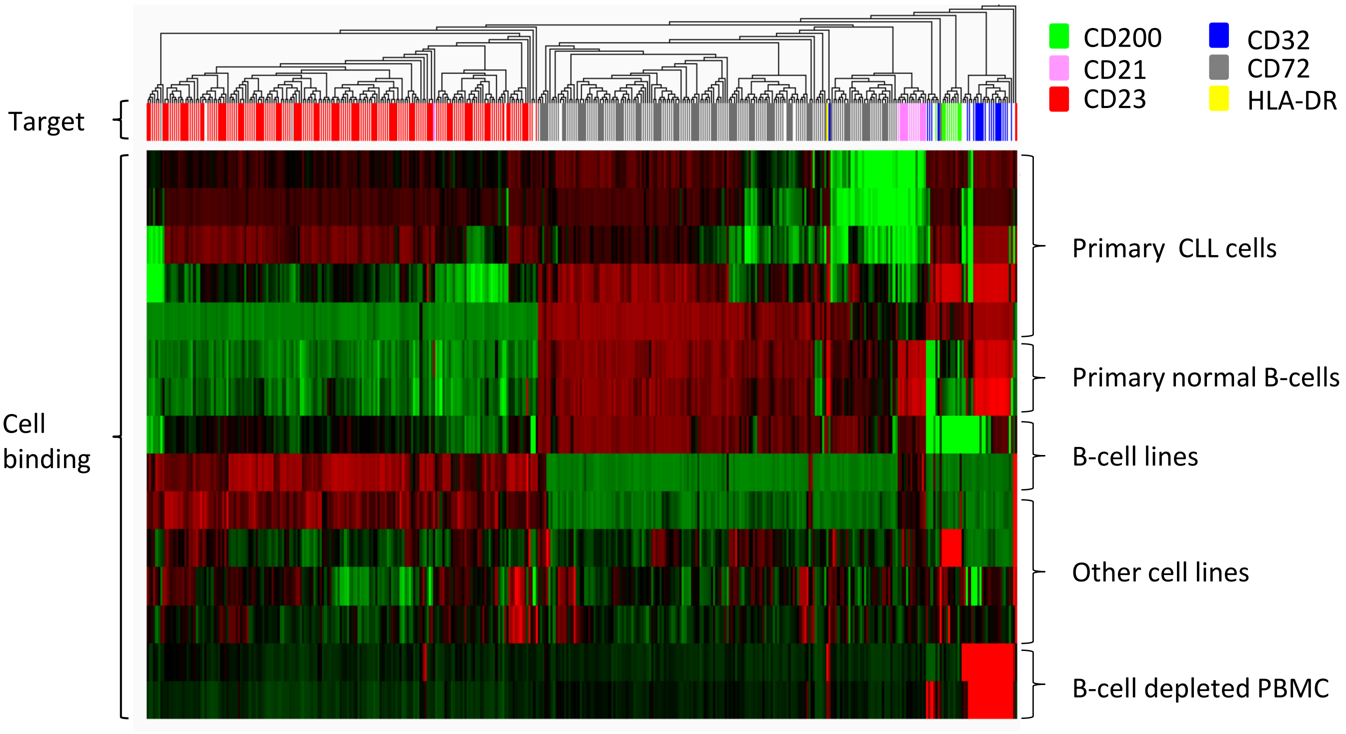

Supplementary Figure 3. Correlation of target identity and cell binding specificity

The cell binding specificity heat map is the same as shown in Figure 2c. Clones were color-coded based on relative signal intensities within each cell type where red represents the strongest binding to a particular cell type, green the weakest binding and black in-between. Identified targets are shown by a separate color coding on top of the heat map.

Supplementary Figure 4

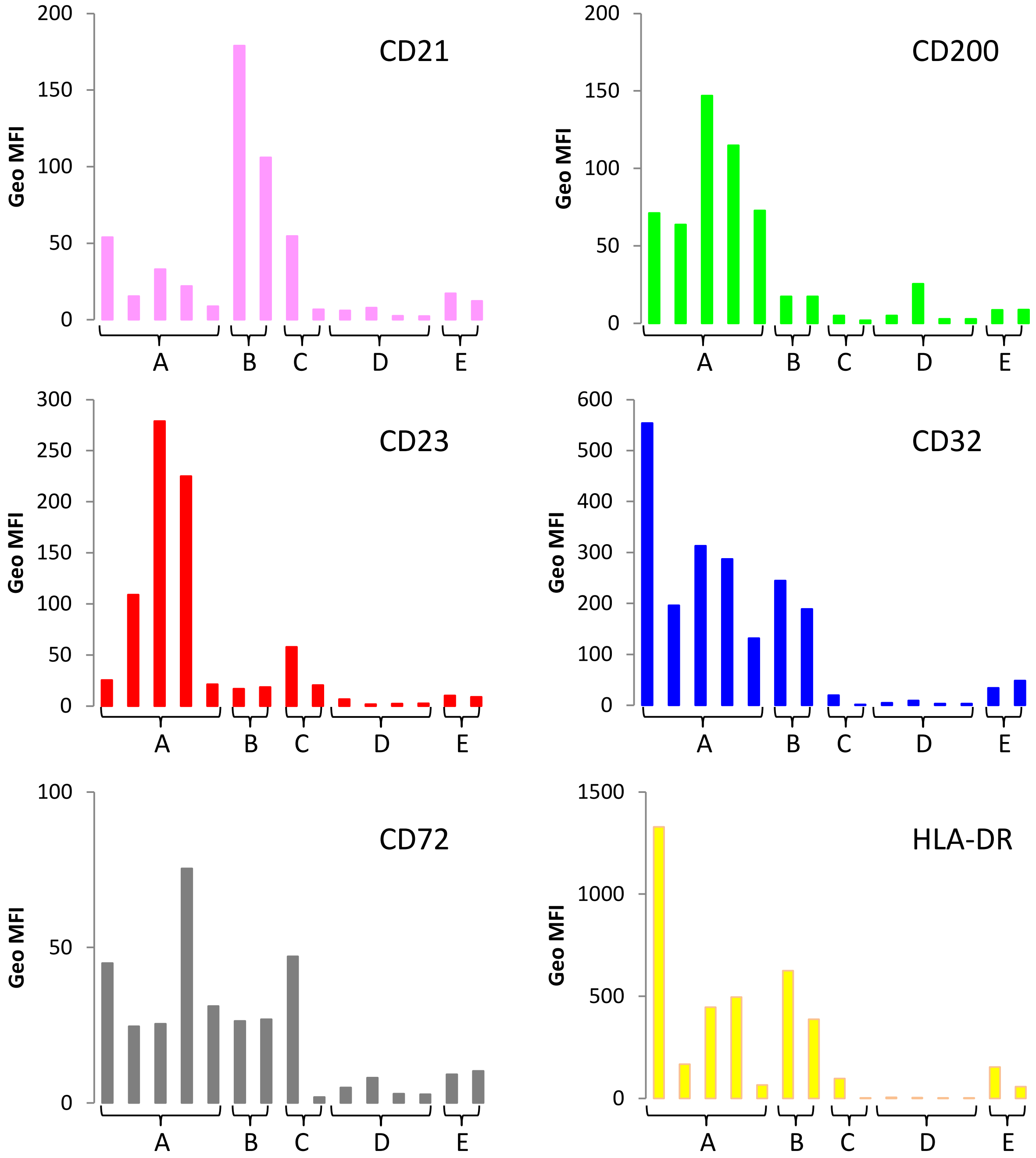

A) Primary CLL cells from 5 patients
B) Primary B-cells from two donors
C) B-cell lines, Raji and RPMI8226
D) Other cell lines, DU145, Lovo, MCF-7 and HS-5
E) B-cell depleted PBMC from two donors

Supplementary Figure 4. Representative cell binding profiles for antibodies with various specificities
The cell binding intensity in flow cytometry is shown for one representative scFv specific for each of the identified targets (CD21, CD23, CD72, CD200, CD32 and HLA-DR). scFvs were selected to represent the identified cell specificity clusters in Supplementary Figure 3. Data are the same that was used to construct the cell binding heat map in Figure 2c.

Supplementary Figure 5

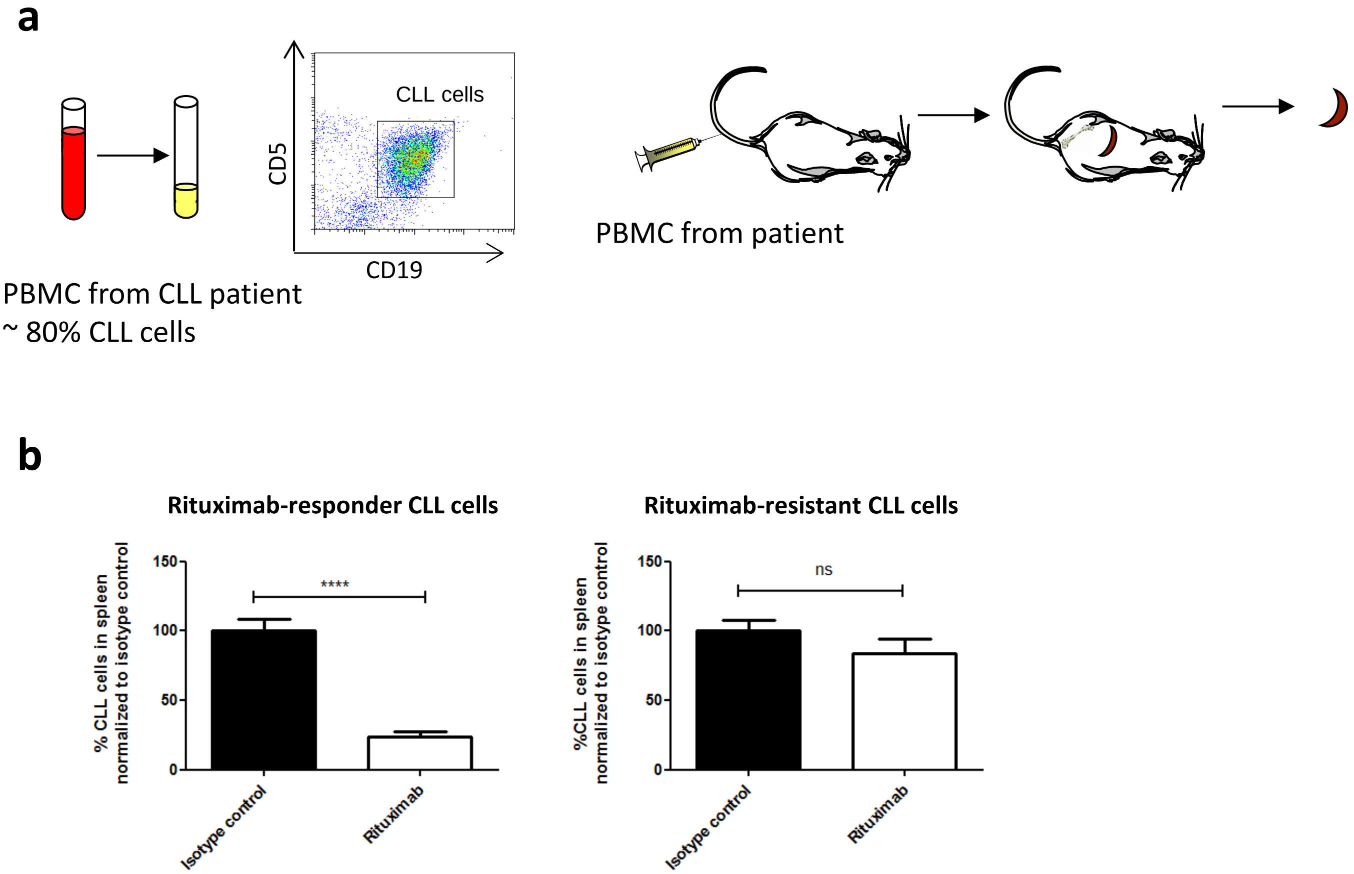

Supplementary Figure 5. Generation of a PDX model and screening of antibodies in vivo

(a) Schematic overview of the PDX model used for in vivo screening. PBMC from a CLL patient, containing mainly CD5+, CD19+ CLL cells, was injected i.v. into irradiated NOD/NOG-SCID mice at day 0. After injection the CLL cells home to lymphoid organs. Antibody (hIgG1), 10mg/kg, was administrated on day 4 and 6 before the mice were sacrificed on day 8. The spleen was collected and the fraction of CLL cells was analyzed by flow cytometry and normalized to the isotype control. (b) Fraction of CLL cells after rituximab treatment, data is normalized to isotype control and show mean with SEM ((rituximab-responder n=20 mice for both isotype control and rituximab), rituximab-resistant n=16 mice for isotype control and n=17 for rituximab). For statistical analysis a t-test using GraphPad Prism was done.

Supplementary Table 1

Methods used for target deconvolution and confirmation of identified targets

	Target deconvolution			Target confirmation	
**Target**	**Retrogenix**	**IP+MS**	**siRNA**	**ELISA**	**FACS block**
**CD21**	NA	+	NA	+	+
**CD23**	+	+	+	+	+
**CD32**	+	+	+	+	+
**CD72**	NA	+	-	+	+
**CD200**	+	+	-	+	+
**HLA-DR**	-	+	-	+	+

+ = identified with this method
- = not identifed with this method
NA = not analyzed

Supplementary Table 2

Gene and protein expression levels on CLL cells and CD19- PBMC from healthy donors for identified targets

	Protein expression			mRNA expression
**Target**	**No of receptors/ CLL cell (n=9)**	**No of receptors/ CD19- PBMC cell (n=6)**	**Target/non-target ratio**	**Target/non-target ratio**
**CD21**	4 000	400[1]	10	11
**CD23**	50 000	300[1]	167	36
**CD32**	50 000	2 000	25	8
**CD72**	8 000	200[1]	40	8
**CD200**	30 000	1 000	25	49
**HLA-DR**	90 000*	2 000	45	7

* n=8

1) Receptor no close to background level (175 obtained with isotype control)

Supplementary Material and Methods

Cells

Buffy coats from healthy donors and blood from CLL patients (Lund University Hospital) were collected in accordance with ethical permission from the Ethics Committee of Skåne University Hospital. PBMC from healthy donors were prepared from buffy coats by gradient density centrifugation (Ficoll Paque PLUS, GE Healthcare). When stated, B-cells were depleted using CD19 Microbeads (Miltenyi Biotec) according to the manufacturer's instructions. PBMC, containing 80-95% B-cells, from CLL patients' blood were also purified by gradient density centrifugation and frozen (10% DMSO in FBS) unless otherwise stated (in the following text denoted "CLL cells"). Cell lines (Raji: Burkitt's Lymphoma, RPMI 8226: multiple myeloma, DU 145: prostate cancer, LoVo: colorectal adenocarcinoma*,* MCF7: breast adenocarcinoma and HS-5: immortalized bone marrow stromal cells) used in the binding pattern analysis were obtained from ATCC and cultured according to the suppliers' instructions.

Isolation of CLL-specific antibodies

Target cells, CLL cells from 10 patients, were pooled and B-cells were labelled with CD19 Microbeads (Miltenyi Biotec) according to the manufacturer's instructions, mixed with an excess of non-target cells, comprising PBMC from 5 healthy donors (with or without prior B cell depletion), and incubated with the n-CoDeR® single-chain fragment variable, scFv, phage display library (BioInvent International) on ice for 2h on a rocking platform. To allow CLL cell separation, the cell-phage mixture was loaded on a MACS column followed by extensive washing to remove any un-bound phages and non-target cells. Finally CLL cells were eluted from the column and phages bound to CLL cells were recovered using digestion with trypsin (Sigma-Aldrich) followed by inactivation using aprotinin (Sigma-Aldrich). Exponentially growing E. coli HB101F' (in-house constructed from E. coli HB101, Thermo Fisher Scientific) was infected with the eluted phages, spread on selective agar plates and

incubated overnight at +30°C. Colonies were pooled and cultivated to produce an amplified phage stock using R408 (Agilent technologies) as helper phage. Amplified phages were then used for another round of panning as described above. After two or three pannings, phagmid-DNA was purified (Miniprep kit, Qiagen, 27104) according to the manufacturer's instructions and the genes encoding scFv fragment were digested from the phagmid vector and ligated into a protein expression vector (in-house constructed). Chemically competent E. coli Top 10 (Thermo Fisher Scientific) was transformed and spread on selective agar plates. Single colonies were picked (Qbot, Molecular Devices) and used for production of individual soluble scFv carrying a 3xFLAG and a 6xHis tag.

Binding analysis of antibodies

Soluble scFv was produced in microtiter plates and supernatants were filtered (0.45µm filter plates, Millipore) before usage. For binding analysis, cryopreserved cells were thawed, washed and blocked with human IgG before addition to flow cytometry or FMAT plates. For flow cytometry, 100 000 cells/well in 25µl PBS with 0.5% BSA were added to 96-well plates followed by addition of 25µl/well of filtered scFv supernatant. scFv was left to bind for 1 h at +4°C followed by washing. Bound scFv was detected using mouse anti-His (R&D Systems, MAB050) and anti-mouse-IgG-APC (Jackson ImmunoResearch, 115-136-146) and cells were analyzed using a FACSCalibur instrument (BD Biosciences). For FMAT analysis, a homogenous assay was run. Cells were seeded in PBS with 0.5% BSA at 2500/10 000 cells per well to 384/ 96-well FMAT plates (Thermo Fisher Scientific), followed by addition of 10µl/well of scFv supernatant and a mixture of mouse anti-His (R&D Systems, MAB050) and anti-mouse-IgG-APC (Jackson ImmunoResearch, 115-136-146) in a total volume of 70µl/well. Plates were incubated for 10h at room temperature before reading using the 8200 cellular detection system (Thermo Fisher Scientific). In analysis of flow cytometry data, scFvs with a geometric mean fluorescence signal at least 2 times above a negative control scFv on the CLL cells and a ratio between CLL and B-cell depleted PBMC from healthy donors above 2 were selected as CLL specific. In the homogenous FMAT assay, CLL-specific scFvs were defined to have at least 3 times more counts

than a negative control scFv on the CLL cells and less or similar number of counts as the negative control scFv on B-cell depleted PBMC from healthy donors.

CLL-specific scFvs were analyzed by Sanger DNA-sequencing and unique clones were re-produced, as described above, and used for binding pattern analysis. Primary CLL cells (5 patients), primary normal B-cells (CD19+ cells, 2 healthy donors), B-cell depleted PBMC (CD19- cells, 2 healthy donors), B-cell lines (Raji and RPMI8226) and other cell lines (DU145, Lovo, MCF-7 and HS-5) were stained with scFv and binding detected as described above for flow cytometry. Hierarchical clustering of scFv based on binding pattern was done using Qlucore Omix Explorer (Qlucore, Plot type Heat and Normalization Mean=0, Var=0) based on percent positive cells (after subtraction of values for an isotype control).

Production of IgG

Antibody genes, VH and VL, were PCR amplified and inserted into an in-house developed expression vector containing genes encoding the heavy and light chain constant regions of a human IgG1 antibody. IgG expression vectors were prepared from E. coli using a kit (Qiagen, 27291) according to the manufacturer's instructions. Suspension adapted HEK 293 EBNA (Thermo Fisher Scientific) was diluted in Freestyle293 medium (Thermo Fisher Scientific) supplemented with 10% Pluronic F-68 (Thermo Fisher Scientific), seeded into 24-well plates, and transiently transfected using PEI (Polyscience Inc). The cells were incubated for 4h, +37°C, 8% CO_2, 300rpm before addition of feed, UltraPep™ Soy (Sheffield Bio-Science), followed by additional 6 days of incubation before harvest. Harvested cell supernatants were incubated with MabSelect resin (GE Healthcare) to allow antibody binding, followed by transfer of the resin to a 96-well filter plate (Thermo Fisher Scientific) for washing and finally elution of purified antibodies using 100mM Glycine, pH 2.8. Purified hIgG1 antibodies were dialyzed (DispoDialyser, Harvard Apparatus) to PBS buffer before concentration measurement, A_{280} (UV Star plate, Greiner), in a plate reader (Tecan Infinite F500, Tecan).

Programmed cell death, PCD

Antibodies (hIgG1) were added to fresh CLL cells, 100 000 cells/well, at 10μg/ml, cross linked using a F(ab)'2 goat anti-human-Fc specific antibody (Jackson ImmunoResearch, 109-006-098) at a 1:5 molar ratio and incubated in RPMI medium supplemented with 10% Ultra-Low IgG FBS, 10mM HEPES and 1mM sodium pyruvate (all from Thermo Fisher Scientific), at +37°C, 5% CO_2 over-night. Cells were stained with Annexin V-APC and SYTOX Green (both from Thermo Fisher Scientific) and analyzed by flow cytometry (FACSCalibur, BD Biosciences). The high concentration of IgG was used to minimize the effect of affinity variations between antibodies both here and in the ADCC assay described below. Antibodies were tested as duplicate samples within each experiment and in several independent experiments on CLL cells from 2-5 patients. For evaluation, the fraction of Annexin V^+ cells (including both SYTOX Green positive and negative cells) compared to all cells was calculated, for normalization the average of the duplicate samples was calculated followed by subtraction of the corresponding value for the isotype control antibody.

Antibody dependent cellular cytotoxicity, ADCC

Antibodies (hIgG1) were added to fresh CLL cells, 50 000 cells/well, at 10μg/ml and left to bind for 30 min at +4°C before addition of a NK cell line expressing green fluorescent protein (NK-92.05 GFP-CD16 176V, Conkwest) at a 2:1 NK:CLL cell ratio. The antibody cell mixture was incubated for 4 h in RPMI medium supplemented with 10% Ultra-Low IgG FBS, 10mM HEPES and 1mM sodium pyruvate (all from Thermo Fisher Scientific), at +37°C, 5% CO_2. Cells were stained with SYTOX Red (Thermo Fisher Scientific) and analyzed by flow cytometry (FACSCalibur, BD Biosciences). Antibodies were tested as triplicate samples within each experiment and in several independent experiments on CLL cells from 2-5 patients. The fraction of dead target cells was determined as SYTOX Red^+, GFP^- cells divided with all target cells, GFP^-. For normalization the fraction of dead cells for the isotype control antibody was subtracted from the average of the triplicate samples.

Target deconvolution

Three different techniques were used for target deconvolution of antibodies. 1) Antibodies (hIgG1) were analyzed for binding to transfected HEK cells overexpressing individual human cell surface proteins in an array format (Retrogenix). 2) 150 genes with a high gene expression ratio CLL versus B-cell depleted PBMC from healthy donors (determined by micro array analysis, Affymetrix, data not shown) were selected for siRNA knockdown experiments. A siRNA library was custom made (Sigma-Aldrich) containing 3 individual siRNA per target gene. Two mantel cell lymphoma cell lines Z138 and GRANTA-519 were selected for nucleofection using an Amaxa 4D-Nucleofector (Lonza) according to the manufacturer's protocol. Antibody binding to transfected and control cells was assessed 48h post transfection using AF647-labelled hIgG1 or non-labelled hIgG1 and a secondary anti-human-IgG-APC antibody (Jackson ImmunoResearch, 109-136-098) and analyzed by flow cytometry (HTFC, IntelliCyt). Specificity was determined as loss of antibody binding after transfection with the corresponding siRNA. 3) Immunoprecipitation was performed with cell lysate from cells that bound the antibody of interest. Cell lysate was prepared by addition of 1% NP40 (Thermo Fisher Scientific) in 50mM TrisHCl (BioLogicals), 150mM NaCl (Merck) and 1mM HEPES (Thermo Fisher Scientific), pH 7.7, to the cells ($1x10^8$) and incubation on ice for 10min followed by centrifugation at 12000g, +4°C for 20min to remove the nuclei. IgG was captured on protein G beads (Thermo Fisher Scientific) followed by washing and incubation with cell lysate for at least 1h at +4°C with end over end rotation. Beads were washed and the target-antibody mixture was eluted in NU-PAGE buffer with 50mM DTT (both from Thermo Fisher Scientific) for 5 min at 90°C and loaded on a precast SDS-PAGE gel (NP0321BPX, Thermo Fisher Scientific) which was run for 1h at 170V. The gel was stained with Coomassie blue (Thermo Fisher Scientific), according to the manufacturer's instructions, and gel pieces with detected protein bands were used for in gel trypsin digestion followed by mass spectrometry (MALDI-TOF MS Bruker Daltonics microflex instrument) analysis.

To confirm antibody specificity, antibodies were analyzed for binding to proteins in ELISA. Proteins, (CD72 from R&D Systems, 5405-CD, CD32b produced in-house, CD200, CD21 and CD23 from Sino Biological, 10886-H08H, 10811-H08H, 10261-H07H and HLA-DR from ProSpec, pro-1889 and pro-1888) were coated to ELISA plates overnight. Antibodies (hIgG1) were diluted to 20µg/ml followed by 2 times dilution and added to the coated and washed plates. Bound IgG was detected by an anti-human-F(ab)'2-HRP antibody (Jackson ImmunoResearch, 109-036-088) followed by a luminescent substrate (37070, Thermo Fisher Scientific) and reading (Tecan Ultra, Tecan). Antibody specificity was further confirmed by flow cytometry. Antibodies (hIgG1 or scFv) were analyzed for binding to Raji or CLL cells with or without pre-blocking with a polyclonal antibody (rabbit anti-CD72 from Cloud Clone Corp. PAB261Hu01, rabbit anti-CD32, rabbit anti-CD21, rabbit anti-CD23 and rabbit anti-CD200 (all from Sino Biological 10259-RP03, 10811-RP02, 10261-T48, 10886-T32) and human monoclonal IgG1 anti-HLA-DR (in house developed[1]). Binding of the antibodies was detected with a fluorophore-labelled secondary antibody (anti-human-IgG-APC, Jackson ImmunoResearch, 109-136-098 or anti-His, R&D Systems, MAB050 labelled in-house with AF647) and analyzed by flow cytometry (HTFC, IntelliCyt).

Receptor number determination

Receptor numbers per CLL cells and PBMC from healthy donors were determined using quantification beads (Bang Laboratories) according to the manufacturer's instructions. Briefly, labelled commercially available antibodies (anti-CD32-APC, anti-HLA-DR-APC and anti-CD23-APC from BD Biosciences 559769, 559866, 558690, anti-CD200-APC and anti-CD21-APC from BioLegend 329208, 354906 and anti-CD72-eFlour660 from eBioScience 50-0729) were titrated for binding to CLL cells and PBMC from healthy donors. Antibodies were then tested at binding saturated concentrations on CLL cells from 9 patients and PBMC from 6 donors, with simultaneous staining of quantification beads, analyzed in flow cytometry (FACS Verse, BD Biosciences) and the number of receptors was calculated according to the supplier's instructions. PBMC were in addition stained with

anti-CD19-PE (BD Biosciences, 555413) and anti-CD45-perCP-Cy5.5 (BD Biosciences, 552724) and non-target cells were defined as CD19-, CD45+ cells.

In vivo screening of antibodies

Mice were maintained in local facilities according to guidelines and experiments were performed in agreement with ethical permissions (M53-14) from the local ethical committee (Malmö Lund Animal Ethics Committee). Fresh CLL cells (6-10x10^7 cells/mice) were injected i.v. into irradiated NOG or NOD-SCID mice (Taconic and Charles River Laboratories) on day 0. Antibody, 10mg/kg, was administrated on day 4 and 6 and the mice were sacrificed on day 8. The spleen was collected and the fraction of CLL cells, defined as human $CD45^+$ (BD Biosciences, 555485), was determined by flow cytometry. Statistical analysis (1-way ANOVA) was performed using GraphPad Prism (Graphpad Software).

Reference

1. Fransson, J., Tornberg, U.C., Borrebaeck, C.A., Carlsson, R. & Frendeus, B. Rapid induction of apoptosis in B-cell lymphoma by functionally isolated human antibodies. *Int J Cancer* **119**, 349-358 (2006).
